# Value of MRI - T2 Mapping to Differentiate Clinically Significant Prostate Cancer

**DOI:** 10.1007/s10278-024-01150-6

**Published:** 2024-06-26

**Authors:** Andreas Michael Bucher, Jan Egger, Julia Dietz, Ralph Strecker, Tom Hilbert, Eric Frodl, Mike Wenzel, Tobias Penzkofer, Bernd Hamm, Felix KH Chun, Thomas Vogl, Jens Kleesiek, Martin Beeres

**Affiliations:** 1 Institute for Diagnostic and Interventional Radiology, Goethe University Frankfurt, University Hospital Frankfurt, Theodor-Stern Kai 7, 60590 Frankfurt, Germany; 2 Institute for AI in Medicine, University Hospital Essen, Girardetstraße 2, 45131 Essen, Germany; 3 Siemens Healthineers AG, (EMEA Scientific Partnerships), Henkestraße 127, 91052 Erlangen, Germany; 4 Advanced Clinical Imaging Technology, Siemens Healthineers International AG, EPFL, QI E, 1015 Lausanne, Lausanne Switzerland; 5 Department of Radiology, Lausanne University Hospital and University of Lausanne, Lausanne, Switzerland; 6 LTS5, École Polytechnique Fédérale de Lausanne (EPFL), Lausanne, Switzerland; 7 Department of Urology, Goethe University Hospital, Goethe University Frankfurt, Frankfurt, Germany, Theodor-Stern Kai 7, 60590 Frankfurt, Germany; 8 Department of Radiology, Charité - Universitätsmedizin Berlin, Charitéplatz 1, 10117 Berlin, Germany; 9 Departement of Neuroradiology, University-Hospital of Giessen and Marburg Campus Marburg, Baldingerstraße 1, 35043 Marburg, Germany; 10 Berlin Institute of Health, Berlin, Germany; 11 Institute for AI in Medicine, University Hospital Essen, Girardetstraße 2, 45131 Essen, Germany; 12 Department of Physics, TU Dortmund University, Otto-Hahn-Straße 4, 44227 Dortmund, Germany; 13 Cancer Research Center Cologne Essen (CCCE), West German Cancer Center Essen (WTZ), 45122 Essen, Germany; 14 German Cancer Research Center (DKFZ), Partner site University Hospital Essen, German Cancer Consortium (DKTK), 45122 Essen, Germany; 15 Medical Faculty, University of Duisburg-Essen, 45122 Essen, Germany

**Keywords:** Multiparametric prostate MRI, T2 mapping, Prostate cancer, Quantitative imaging

## Abstract

Standardized reporting of multiparametric prostate MRI (mpMRI) is widespread and follows international standards (Pi-RADS). However, quantitative measurements from mpMRI are not widely comparable. Although T2 mapping sequences can provide repeatable quantitative image measurements and extract reliable imaging biomarkers from mpMRI, they are often time-consuming. We therefore investigated the value of quantitative measurements on a highly accelerated T2 mapping sequence, in order to establish a threshold to differentiate benign from malignant lesions. For this purpose, we evaluated a novel, highly accelerated T2 mapping research sequence that enables high-resolution image acquisition with short acquisition times in everyday clinical practice. In this retrospective single-center study, we included 54 patients with clinically indicated MRI of the prostate and biopsy-confirmed carcinoma (n = 37) or exclusion of carcinoma (n = 17). All patients had received a standard of care biopsy of the prostate, results of which were used to confirm or exclude presence of malignant lesions. We used the linear mixed-effects model-fit by REML to determine the difference between mean values of cancerous tissue and healthy tissue. We found good differentiation between malignant lesions and normal appearing tissue in the peripheral zone based on the mean T2 value. Specifically, the mean T2 value for tissue without malignant lesions was (151.7 ms [95% CI: 146.9–156.5 ms] compared to 80.9 ms for malignant lesions [95% CI: 67.9–79.1 ms]; p < 0.001). Based on this assessment, a limit of 109.2 ms is suggested. Aditionally, a significant correlation was observed between T2 values of the peripheral zone and PI-RADS scores (p = 0.0194). However, no correlation was found between the Gleason Score and the T2 relaxation time. Using REML, we found a difference of -82.7 ms in mean values between cancerous tissue and healthy tissue. We established a cut-off-value of 109.2 ms to accurately differentiate between malignant and non-malignant prostate regions. The addition of T2 mapping sequences to routine imaging could benefit automated lesion detection and facilitate contrast-free multiparametric MRI of the prostate.

**Supplementary Information:**

The online version contains supplementary material available at 10.1007/s10278-024-01150-6.

## Introduction

Prostate cancer (pCA) is the second most common solid tumor in men worldwide and has the highest age-standardized rate (ASR) in Northern Europe at 83 per 100,000. The lifetime cumulative risk to develop a pCA is 3.9% [1].

Multiparametric MRI (mpMRI) is the preferred imaging modality for prostate cancer and includes T1-weighted, T2-weighted (T2w), diffusion-weighted (DWI), and dynamic contrast-enhanced (DCE) sequences [2, 3]. MpMRI complements digital rectal examination (DRE), serum-PSA measurement, transrectal ultrasound-guided (TRUS) in imaging. So far, the mpMRI has generally shown high sensitivity but low specificity in diagnosing pCA [4].

Standardized reporting among urologists, radiologists and oncologists is crucial for the diagnosis and treatment planning of pCA. Although widespread and well-established, scoring of prostate lesions using the Prostate Imaging-Reporting and Data System (PI-RADS) is subjective by nature. in particular, non-experienced radiologists may find it challenging to differentiate between tumor-free and tumor-containing tissue in the prostate [5]. However, standard mpMRI acquisitions do not regularly provide quantitative image measurements, resulting in subjective or semi-objective interpretation and poor reproducibility of quantitative measurements on follow-up examinations.

The T2w sequence is particularly relevant for anatomical assessment of the prostate. Local variations in radiofrequency inhomogeneities of the transmitting and receiving coil make T2w values generally suitable as a qualitative measure. T2 mapping (T2_M_) values, on the other hand, can be processed as a quantitative parameter since they are based on multiple echo times, and thus reflect the relaxation time of the protons independent of their relative position to the coil [3]. However, the unfavorable resolution to acquisition time ratio has impeded the wide-spread adoption of this technique into standard clinical protocols [3].

Previous investigations have shown that T2_M_-derived measurements can detect malignant prostate lesions in standardized mpMRI [3, 6–8]. In this analysis, we evaluated the value of the latest-generation quantitative MR data acquisition methods in detecting and characterizing prostate lesions in the peripheral zone [9]. A research application was utilized for fast T2_M_ with high spatial resolution, supporting parallel imaging and model-based reconstruction [9]. The technical novelty of this technique allows for high acceleration, resulting in acquisition times that are acceptable in clinical routine.

We therefore evaluated the mean relaxation time of malignant lesions compared to benign lesions and tumor-free prostate regions in different age groups. Additionally, the study aimed to determine if radiologists could use the highly accelerated T2_M_ sequence to more easily distinguish malignant from healthy tissue. The study derived a threshold value to help classify suspicious prostate lesions as malignant or non-malignant.

## Material and Methods

### Patients

**Fig. 1:**
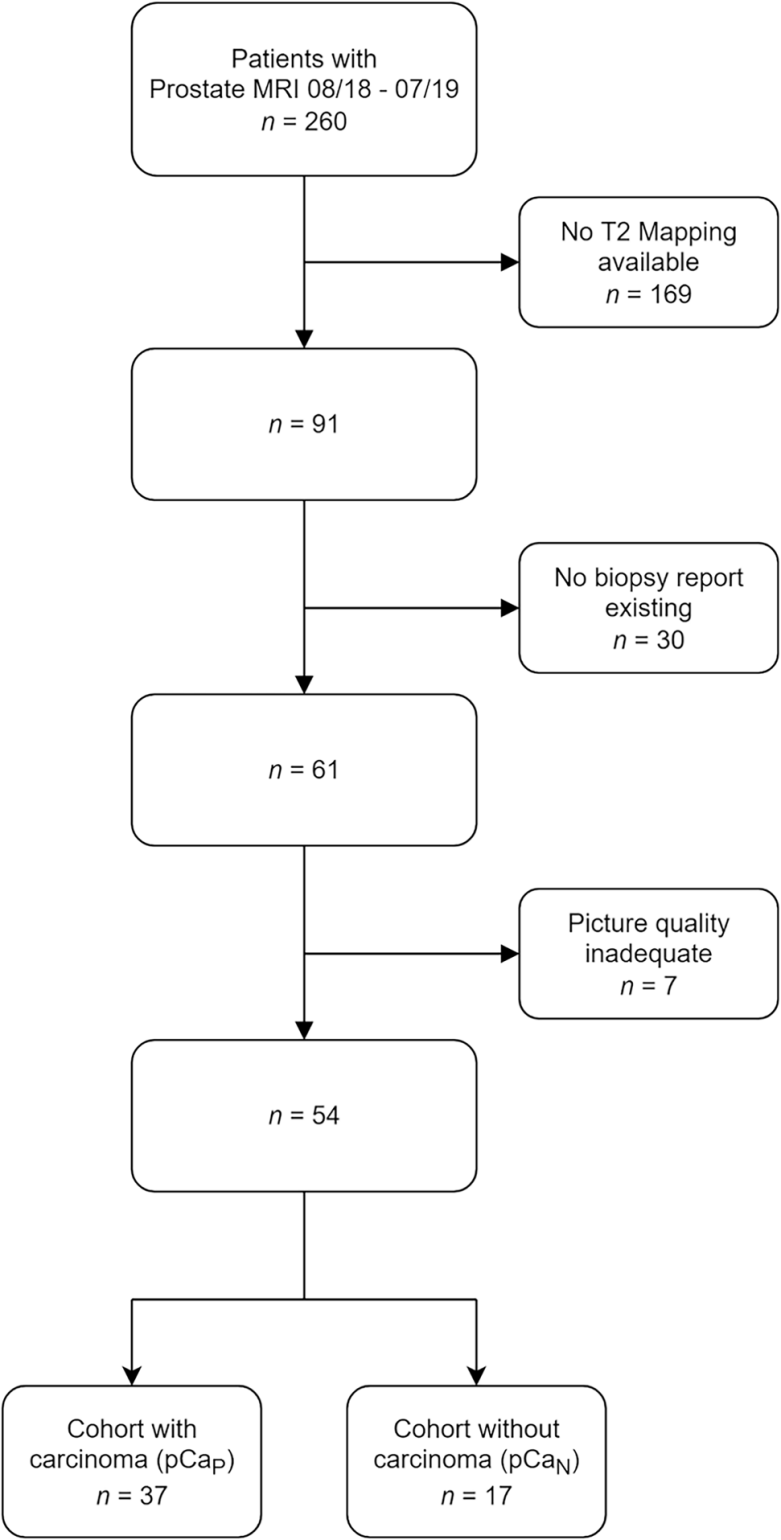
Flowchart of cohort composition. For this retrospective analysis patients were divided into two arms, according to biopsy results. All patients had received a systematic biopsy, which was considered as ground truth for the exclusion or confirmation of prostate cancer

We conducted a retrospective case-control study, approved by the Institutional Review Board (IRB No. 19-299). Cases were identified by an independent investigator by querying the hospital's Picture Archiving and Communication System (PACS) for prostate MRIs between 08/2018 and 07/2019. The study’s inclusion criteria required an examination to exclude prostate carcinoma. Patients who had received a T2_M_ sequence as part of the mpMRI protocol with total coverage of the prostate volume during this period were included (acquisition parameters are included in the Supplementary material). Of 260 patients, 91 met these inclusion criteria (Fig. 1).

**Fig. 2:**
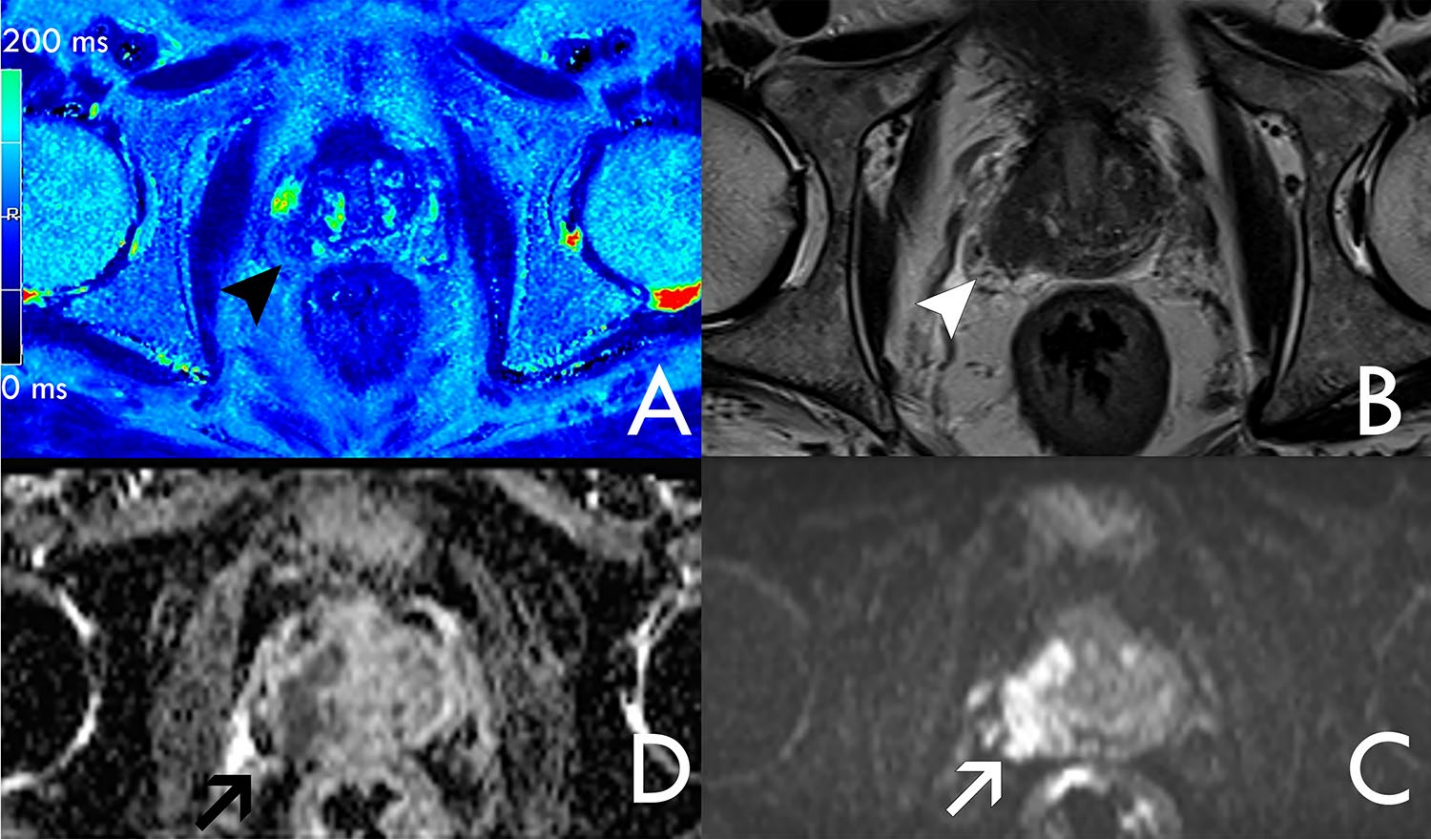
Confirmed prostate cancer of the right peripheral zone. Coregistration of the sections from T2_M_ (**A** the color scale on the left image border is ranging from 0 to 200 ms), T2w (**B**), DWI b1000 (**C**) and ADC (**D**) sequences for one case of the pCA_p_ group. The malignant lesion of the right peripheral zone is marked by an arrowhead in T2_M_ where darker values correspond to lower relaxation times. In the corresponding region of the T2w decreased signal intensity can be observed in the posterolateral aspect of the right peripheral prostate zone (arrowhead). From ADC and DWI, a diffusion restriction is visible, demonstrating infiltration of the right transitional zone (arrow). The figure includes a color legend on the right border indicating relaxation times per voxel. Relaxation times larger than 200 ms are displayed in red

The data from the clinical database concerning the patients and their MRI were recorded and transferred to our study database (including the population parameters age, examination date, findings on mpMRI, PIRADS score) (Fig. 2).

From the hospital information system, we recorded whether the patient had undergone previous surgery, biopsy, or ablation or embolisation of the prostate, as well as the presence of prostatitis. The final cohort included only patients who had not undergone any interventions on the prostate and had a prostate biopsy subsequent to mpMRI. Biopsy was defined as a systematic, standard-of-care biopsy. For these, pathology reports had to be available in the hospital data system and specify results per prostate region (n = 61).

The remaining group was further defined based on data quality criteria. Exclusion criteria included poor image quality due to relevant motion artifacts, incomplete or non-displayable T2_M_ sequences, and unavailable or incomplete biopsy reports. Poor image quality was defined as not sufficiently interpretable by an experienced radiologist.

The final study group comprised 54 patients. Of these 37 patients were diagnosed with prostate carcinoma and for 17 cases the presence of prostate carcinoma was excluded. The mean age of the group was 65.5 years (minimum 42, maximum 83 years). Tables 1 and 2 list the median PIRADS scores.

**Table 1:** Population overview

Age (years)	Number of patients included	PI-RADS (Median and Range)	Biopsy correlation available (number of patient)
40–44	1	5	1
45–49	0	-	-
50–54	4	3.5 (3–4)	4
55–59	3	3 (2–5)	3
60–64	15	4 (2–5)	15
65–69	12	4.5 (3–5)	12
70–74	14	4 (2–5)	14
75–79	4	3.5 (3–5)	4
80–84	1	4	1

Median of PI-RADS per patient is presented and the range is included in parentheses

**Table 2:** Population parameters of study and control groups

	***Cohort with carcinoma***	***Cohort without carcinoma***
***Age (y)***	***65.6 (42–83)***	***65.2 (53–77)***
***Gleason Score***	***7.4 (5–10)***	***0***
***PSA level (ng/ml)***	***40.2 (2.9–352.0)***	***14.4 (5.9–33.6)***
***PIRADS***	***4.5 (3–5)***	***2.7 (2–3)***

Values are the mean per parameter, range is in included parentheses, from lowest to highest value

## Procedures and Techniques

### Imaging

MRI imaging was performed for all patients at 3 Tesla (MAGNETOM Prisma^Fit^, Siemens Healthineers, Erlangen, Germany).

**Fig. 3:**
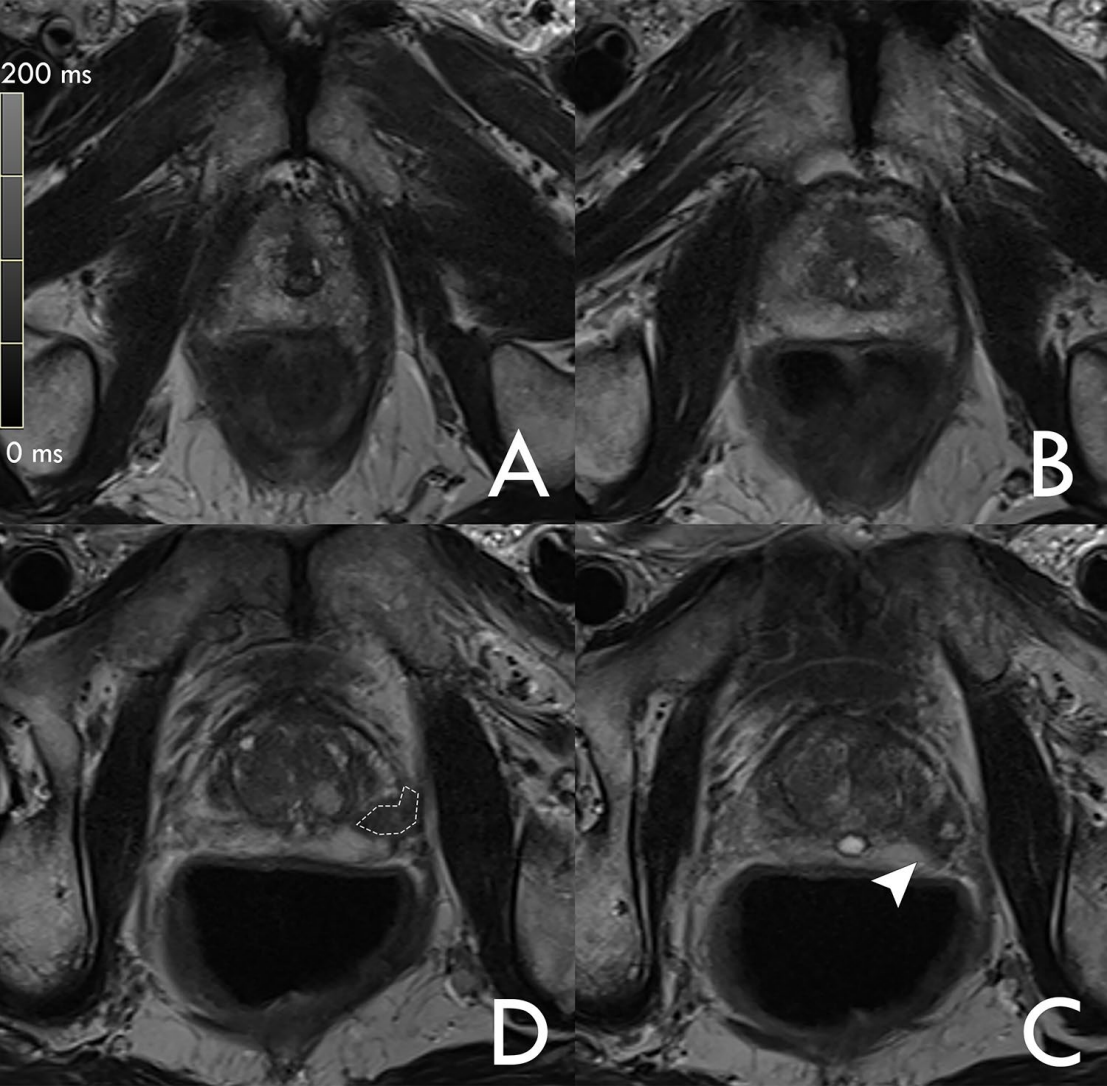
Example of region of interest (ROI) measurement of malignant lesion. T2w sections demonstrating the height of measurements for T2_M_ sequence. Region of interest (ROI) measurements were drawn with the polygonal measurement tool an apical (**A**), midbase (**B**) and base (**C**). Suspicious lesions were additionally measured on the slice with its largest diameter (**D** dotted line). This image represents a confirmed prostate cancer of the left peripheral zone (arrowhead)

The mpMRI protocol included T2w imaging in 3 orientations, Diffusion Weighted Imaging (DWI), dynamic contrast-enhanced T1-weighted (same orientation as axial T2-weighted and DWI) and pre-contrast T2_M_ (0.7 × 0.7 × 3.0 mm^3^, 16 echoes with ΔTE of 10.8 ms, TR 5000 ms, acceleration factor = 10; Fig. 3). Image reconstruction parameters are listed in the Supplementary material. Notably, T2_M_, a research application sequence, “GRAPPATINI”, that accelerates a multi-echo spin-echo sequence is used to achieve acquisition times that are feasible in clinical routine [9]. Particularly in this study, a tenfold acceleration is achieved by combining a twofold GRAPPA acceleration with a fivefold model-based acceleration resulting in an acquisition time of 4:37 min [9, 10]. GRAPPATINI has been evaluated in various body parts including the knee, brain, spine, pancreas, cervix, and prostate [3, 6, 11–15]. The method has been compared to various other methods across organs [13, 16].

### Prostate Biopsy Performance

All patients included in our study underwent a systematic prostate biopsy. Before the biopsy, rectal swabs and/or urine cultures were performed if clinically indicated. A periprostatic local anesthesia was injected under ultrasound-guidance. We took 12 cores, 6 from each prostate lobe, with a length of 15–22 mm. If a targeted fusion biopsy was performed in addition, 2 cores were taken from each suspicious lesion (defined as PIRADS ≥ 3).

Targeted biopsy was performed with a high-end ultrasound-machine (HiVison, Hitachi Medical Systems, Tokyo, Japan) [17–20].

### Interpretation of Biopsy

Imaging abnormalities were defined as confirmed malignancies based on the interpretation of the biopsy results for the corresponding prostate regions. Additionally, the regions of the corresponding lesions were noted and compared with the imaging findings.

### Data Collection

Measurements were performed by an independent assessor trained in the use of PACs measurement tools on a GE Workstation (Universal Viewer, GE, Boston).

For each MRI study, three regions of interest were drawn at representative axial sections: at the level of apex (pTa), mid-base (pTm) and base (pTb). Each ROI was drawn in three different regions per slice, including the right peripheral zone, left peripheral zone and transitional zone. Thus, a minimum of 9 ROIs were drawn per subject.

A differentiation was made between tumor-free (pT) and tumor-containing tissue (pCA). Tumor-containing tissue was defined as PIRADS ≥ 3 and Gleason Score ≠ 0. Prostate tissue without suspicious lesions (as determined by mpMRI and biopsy report) was measured bilaterally in the peripheral zone, unless the zone was completely affected such that a representative ROI could not be set. In this case, only malignant lesion measurements were recorded for this region. The slice with the largest tumor extent was also included to mark a representative ROI for the tumor-tissue (Fig. 3). Image measurements were performed in a structured manner suitable to inexperienced readers.

MRIs were originally marked in the T2w reconstruction of the T2_M_ sequence. All corresponding measurements were mapped to other sequences by table position and synchronization of image stacks. If the automatic matching failed, corrections to the mapping between sequences could be made manually. ROIs were always copied from the first sequence measured to all other corresponding sequences. Therefore, identical ROI shapes and sizes were ensured to match measurements between sequences as closely as possible. Mean and minimum transverse relaxation time (T2) values in each ROI were recorded.

### Measures of Data Validity

To ensure data quality, incomplete data, studies of poor image quality and inconsistent data was rigorously sorted out.

In addition, the ROIs were marked by an independent reader. ROIs were drawn leaving a margin to the edge of each structure to avoid including other tissue. ROIs were made as large as possible to get a representative average value per region. ROIs were not placed in areas containing artifacts.

All measurements and the markings of the ROIs were reviewed by an expert reader (AMB) with 6 years of experience in reading prostate MRI. ROIs were however only redrawn if the desired prostate region was not accurately included, in order to test the suitability of the approach for an inexperienced reader.

### Statistical Tests

Statistical analysis was conducted using commercially available statistical software, including SPSS, version 21.0 (^©^ 1989–2012, IBM, Armonk, NY, USA; MedCalc Software bv, Ostend, Belgium; RStudio, PBC, Boston, MA, USA). The normal distribution was determined using the Shapiro-Wilk test. Categorical variables are presented as percentages, continuous variables as mean ± standard deviation or median and interquartile ranges if the distribution is not normal. The non-parametric were assessed using the Mann-Whitney-U-test. To determine any correlation, the Spearman test was performed. A receiver operating characteristic (ROC) was used to identify the cut-off value that achieved the best balance between sensitivity and specificity. Using a linear mixed-effects model (LMM) fit by Restricted Maximum Likelihood (REML), we assessed the differences in transverse relaxation times (T2) between cancerous and healthy prostate tissues. The model included fixed effects for tissue type (cancerous vs. healthy) and random intercepts for subjects to account for inter-individual variability. P-values less than 0.05 were considered statistically significant.

## Results

### Patient Population

We included all patients between 08/2018 and 07/2019, who received a prostate MRI to rule out a pCA in our institute. Of these 260 patients, in 91 cases T2_M_ were part of the image protocol. Of these 91 remaining patients, 61 received a prostate-biopsy. After excluding patients based on image quality criteria, as assessed by a radiologist with 6 years of experience, a total of 54 patients were included (Fig. 1).

Our patient cohort thus contained 37 patients with biopsy confirmed pCA (pCA_P_) and 17 patients without a pCA (pCA_N_). Mean age was 65.5 years (± 7.7 years, min: 42 years, max 83 years). Patient age was comparable between groups (p = 0.6915; Table 1). Mean PSA level of the pCA_P_ group was 36,1 ng/ml (95% CI: 10.7–61.5 ng/ml).

### Biopsy Results

The biopsy results confirmed pCA in 37 cases. The most common Gleason score was 7. The mean interval between MRI scan and prostate biopsy was 21 days (95% CI: 14–28 days). An example of patients with pCA detected on our MRI protocol is included in Fig. 3.

### mpMRI Results

The PIRADS value was previously determined by radiologists. PIRADS was looked at per lesion in case of several lesions. The average PIRADS was 3.9 (± 1.03), the most frequent PIRADS score was 5 (n = 21; Table 3). T2_M_ values in peripheral zones of healthy tissue but also in diseased tissue were not normally distributed. An overview of the T2_M_ values of the peripheral zone is shown in Table 5.

**Table 3:** PIRADS scores diagnosed by radiologists in the study’s population

PIRADS	number of studies	Confirmed prostate cancer (number of studies)
1	0	0
2	5	0
3	15	3
4	13	13
5	21	21

Number of patients grouped by radiological PI-RADS interpretation prior to biopsy

**Fig. 4:**
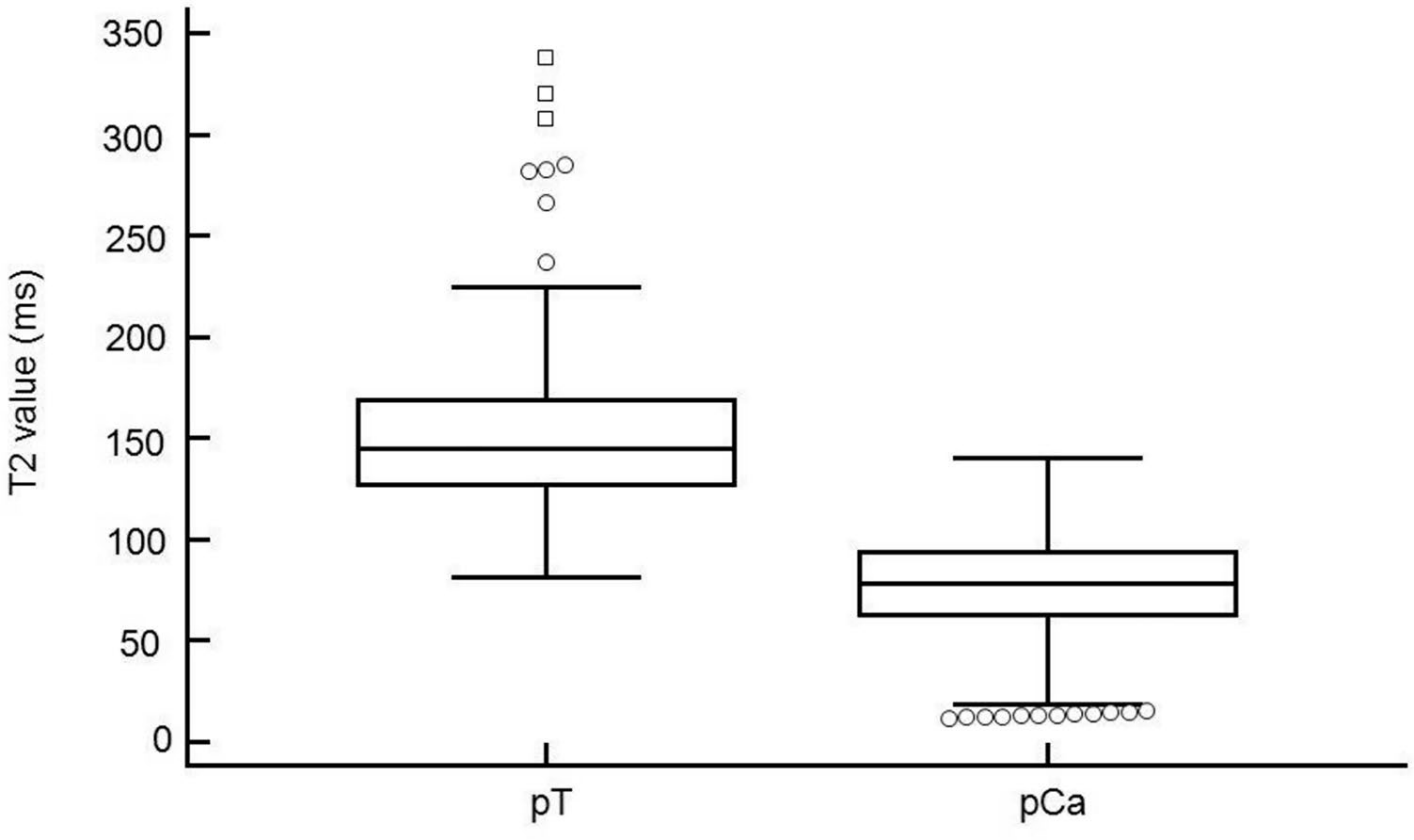
Box-and-whisker plot of measurements from T2 mapping sequence. Relaxation time (ms) in T2 mapping, comparison of healthy tissue (pT) and malignant lesions (pCA); Dots: outside values, squares: far out values, defined as values larger than the upper quartile plus 3 times the interquartile range

The mean relaxation time for healthy tissue was significantly higher at 151.7 ms (95% CI: 146.9–156.5 ms) compared to malignant lesions, which averaged 73.5 ms (95% CI: 67.9–79.1 ms, p < 0.0001; Fig. 4). 95% of the pCA values were below 121.4 ms.

**Fig. 5:**
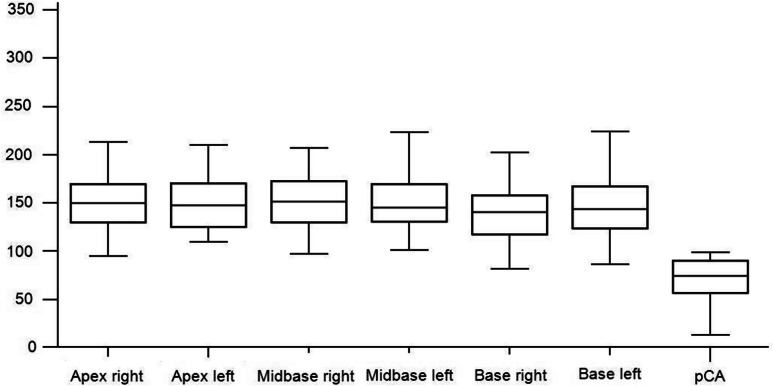
Box-and-whisker plots of T2 mapping measurements per prostate region. T2-Mapping values (ms) are shown grouped by anatomic prostate region of all non malignant tissue measurements. Although there are slight differences in the average relaxation times of prostate tissue per prostate region, there is no significant overlap with malignant lesions measured across all prostate regions. The measurements of peripheral zone regions with cancer affection have been pooled into one group (pCA) for comparison. To represent the reader's differentiation task of comparing benign lesions of any given prostate zone to the average values of malignant lesions, we chose to pool pCA values. Table 5 provides a more detailed comparison of average relaxation times per region for both groups

T2_M_ in different peripheral regions of the prostate did not differ significantly (Table 4, Fig. 5).

**Table 4:** Reference Tissue measurements by region

Region	pT (ms)	pCA (ms)
pTa right	152.4	69.4
pTa left	157.0	78.6
pTa tz	117.5	74.0
pTm right	154.8	76.5
pTm left	153.9	73.6
pTm tz	116.6	87.5
pTb right	142.1	76.9
pTb left	150.6	75.1
pTb tz	115.9	95.8

Mean relaxation time from T2_M_ is listed from a total 54 patients were measured, 360 in the peripheral zone and 165 in the transitional zone

*pT* prostate tissue without suspicious lesions, *pCA* malignant lesion, *pTa* Apex region, *pTm* Midbase region, *pTb* Base region, *tz* Transitional zone, *T2*_*M*_ T2 mapping

**Table 5:** T2 relaxation times of the peripheral zone

Average T2M-Time (ms)												
	Apex right		Apex left		MB right		MB left		Basis right		Basis left	
	PZ	PZ_Ca_	PZ	PZ_Ca_	PZ	PZ_Ca_	PZ	PZ_Ca_	PZ	PZ_Ca_	PZ	PZ_Ca_
Arithmetic Mean	152.4	69.4	156.9	78.6	154.8	76.5	153.9	73.6	142.1	76.9	150.6	75.1
Median	149.7	72.6	147.6	76.5	152.0	77.5	145.4	75.9	140.4	76.9	144.0	83.0
Min	94.8	13.2	109.9	11.9	97.4	13.8	101.5	15.1	81.6	13.1	86.7	13.9
Max	237.2	134.4	337.3	134.4	285.0	140.1	319.8	121.0	266.7	137.5	282.5	99.7
P75	169.3	92.7	107.2	86.8	172.4	97.1	169.2	93.7	158.3	100.6	167.2	93.0

Descriptive statistics of the parameter T2 relaxation times for measurement of the peripheral zone on the three heights used for measurements

*PZ* healthy peripheral zone tissue, *PZCa* peripheral zone carcinoma tissue (confirmed by biopsy), *MB* Mid-Base, *Min* smallest measured value, *Max* largest measured value, *P75* 75th percentile, *ms* all data in milliseconds

The linear mixed-effect model showed that the relaxation time of the mean values in lesions is on average 82.7 ms less than the relaxation time of healthy tissue.

The Spearman test did not reveal any correlation or significant differences between the relaxation time and Gleason score in the diseased tissue. However, peripheral zone T2_M_ values exhibited a correlation with PI-RADS score (p = 0.0194).

**Fig. 6:**
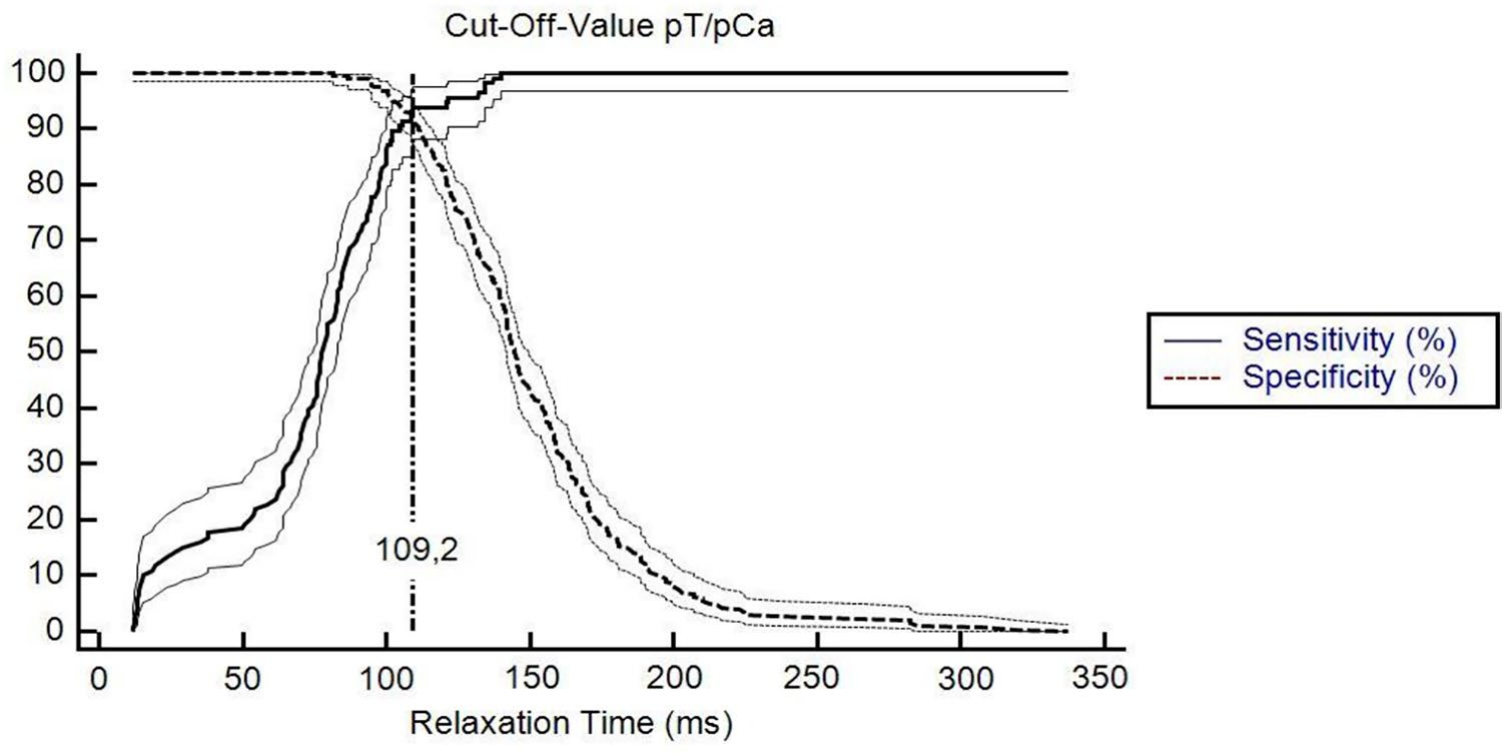
Sensitivity and Specificity in relation to relaxation time of T2 mapping measurements. When considering sensitivity and specificity in relation to relaxation time values, a cut-off-value of 109.2 ms was derived to differentiate malignant from non malignant tissue regions with high accuracy

**Fig. 7:**
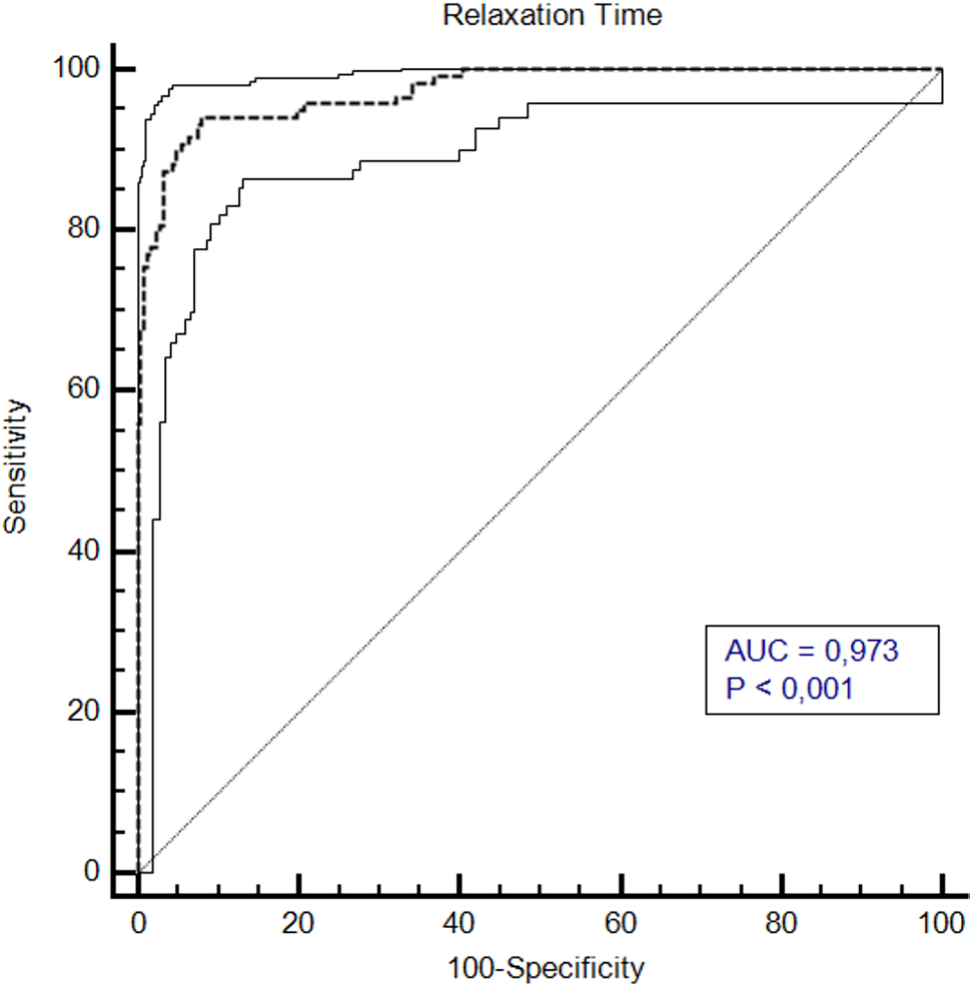
ROC graph of T2 mapping values. In ROC analysis, AUC was found to be 0.973 (95% CI 0.952–0.988) for T2 values (p < 0.001)

**Fig. 8:**
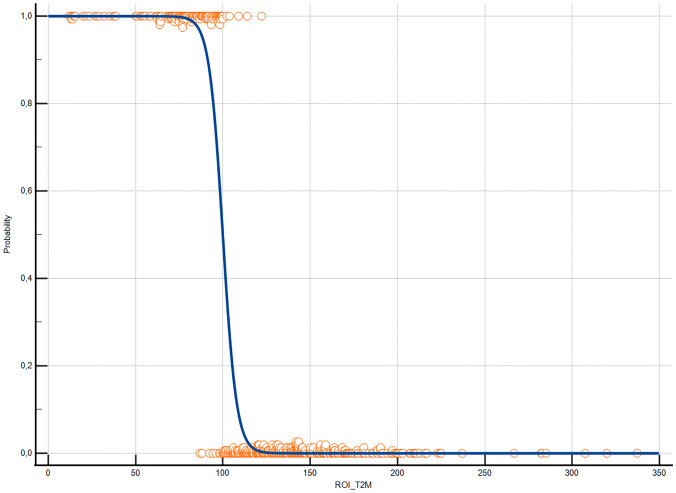
Logistic Regression. Logistic Regression comparison of pooled measurements from ROIs corresponding to regions associated with biopsy confirmation or exclusion of malignant lesions

The ROC analysis of T2_M_-values resulted in an AUC of 0.973 (95% CI [0.951–0.987], p < 0.0001), with a threshold value of ≤ 109.2 ms, (sensitivity: 94.07%, specificity: 92.06%) for the differentiating between pT and pCA (Figs. 6 and 7). We analyzed the relationship of T2_M_-values and the classification into pT and pCA using logistic regression. The logistic regression model was found to be statistically significant (χ2 = 274,2, p-value < 0.0001). The model explained 78,8% (Nagelkerke R2) of the variance in cancer presence and correctly classified 93% of cases. The odds ratio for T2_M_-values was 0,89 (95% CI 0.87 to 0.92), with a coefficient of -0.110 (Standard error: 0.013; p < 0.001; Fig. 8).

## Discussion

One of the challenges radiologists face in differentiating between benign and malignant tissue of the prostate is that conventional T2w and DWI sequences provide qualitative rather than quantitative imaging information. Intra-individual comparisons have shown large heterogeneity in quantitative measurements for these sequences when comparing quantitative measurements in [21]. Diagnosis of prostate cancer remains a subjective process and relies primarily on experienced radiologists [22, 23]. However, our study demonstrates that using quantitative T2_M_ values can aid in distinguishing between healthy tissue and tumors in the peripheral zone of the prostate. The quantitative values for pCA were significantly lower than those for healthy tissue (73.5 ms vs. 151.7 ms). Through ROC analysis, a threshold of 109.2 ms was statistically determined.

Using biopsy confirmation as a reference method, detection of malignant prostate tissue based on quantitative measurements derived from high-resolution T2_M_ sequences can lead to good differentiation of lesions in the peripheral zone.

There was no significant correlation between Gleason Score and tissue measurement. In our study, therefore, the aggressiveness of the lesion cannot be inferred from the measured value.

Our measured T2M values are consistent with those reported in previous studies affirming the reliability of our measurement techniques [3, 7, 24, 25]. Our proposed threshold lies between the two thresholds identified by Yamauchi et al. (99 ms) and Mai et al. (134 ms) [3, 24]. However, our threshold showed a higher sensitivity in our cohort than those two mentioned above. Liu et al. determined reference values in their study on T2_M_ (100 +– 10 ms for malignant lesions, 149 +–32 ms for healthy tissue) [26]. Although our values differ slightly, they both show that the values for malignant lesions are significantly lower than for healthy tissue. Our proposed threshold for the best possible sensitivity and specificity was 109.2 ms, which is close to the values of Liu et al.

As demonstrated in previous studies, a quantitative T2_M_ sequence can provide a repeatable, measurable value for diagnosing malignant-suspicious prostate findings [3, 7, 25]. By utilizing the measured values of the T2_M_, it is possible to differentiate between benign and cancer-related tissue. This method enabled sufficient differentiation of malignant findings to be shown, particularly in the peripheral zone. Wu et al. showed in their study on 31 patients that the specificity and sensitivity are higher if the T2_M_ values are also used additionally to the normal T2w values [27]. Potential uses of quantification in the T2_M_ sequence could be both assessment of the prostate in the case of elevated laboratory parameters (prostate-specific antigen) and follow-up monitoring after therapy to see the response. This is also relevant if the prostate is palpable or if the prostate carcinoma has been histologically confirmed. T2_M_ could help to evaluate the spread of the carcinoma prior to surgery. T2_M_ could thus increase the value of a contrast agent-free mpMRI. In addition, the T2_M_ is also helpful for inexperienced readers since malignant findings appear in a single sequence and do not have to be linked over several sequences. Inexperienced readers usually must assess and compare different sequences of the MRI in order to assess the presence of tumor-free or tumor-containing tissue. The T2_M_ sequence and the significant threshold identified in this study could help readers to assess the occurrence of malignant tissue more reliably by using only one sequence. The suspicion of an assumed malignant change could be confirmed based on measured values. Accelerated T2_M_ could be a feasible addition to standardized multi-parametric MRI of the prostate and could further aid automated lesion detection with quantitative measurements. While we used a simple thresholding method the addition of fingerprinting and pattern recognition approaches could help with characterization of lesions that are more difficult to evaluate, such as transitional zone lesions [28]. In breast cancer diagnostics, for example, studies have already shown that the T2_M_ value of malignant and benign zones in the breast differs [26]. According to Mai et al. the synthetic T2w contrast calculated from the T2_M_ is equal to anatomy and diagnostic accuracy compared to conventional T2w [3]. Therefore, there could be the possibility to use only one sequence (T2_M_) and its synthetic T2w for detection of prostate lesions, replacing the standard T2w acquisition.

This study has some limitations that must be considered when interpreting the results. First, there was not much data acquired with the novel T2_M_ sequence in the clinical database when the study was executed. In some of them, no pathological examination was carried out, so that the results could not be correlated with pathological data. As a result, the number of patients examined is rather small. However, compared to other studies, our study did not examine fewer patients [7, 24, 25].

Furthermore, the exact correlation of the pathology with the image values is only possible to a limited extent. Although the pathological finding confirms e.g. the presence of a carcinoma in a certain zone, this does not mean that the entire zone is affected. It was therefore still necessary for the reader to independently examine the image for the lesion, find it and mark it. Inaccuracies can happen here. To prevent this, different sequences were compared. In particular, the ADC and DWI sequences were helpful in pinpointing the actual lesion. Another limitation is that not all patients had the same type of pathological examination. Prostatectomy is certainly the most accurate. However, this type of histopathology was only performed in a few patients.

Finally, there is the inaccuracy of the measurement of healthy tissue. Here, the examiner chose ROIs in the areas that were designated as non-malignant by the pathology. It cannot be ruled out that in those areas are signal changes too, e.g. because of benign prostatitis, which also leads to signal changes. These benign lesions were not investigated further in our study, in contrast to the study by Hepp et al. [7]. The investigators tried to prevent this by setting the ROIs in healthy tissue as large as possible, paying attention to homogeneity. This minimizes distortion of the results due to possible small areas with changes. There was no significant correlation between Gleason Score and tissue measurement. In our study, therefore, the aggressiveness of the lesion cannot be inferred from the measured value.

## Conclusion

Our investigation confirmed that there are significant differences in the T2_M_ sequence between the relaxation time in healthy prostate tissue and prostate carcinomas in the peripheral zone. We recommend a threshold of 109.2 ms. Our investigation did not show any correlation between the measured value and Gleason Score.Therefore, in the future, the quantitative determination in the T2_M_ sequence could help radiologists to better assess suspicious lesions of the prostate.

## Supplementary Information

Below is the link to the electronic supplementary material.

Supplementary file1 (DOCX 15 KB)

## Data Availability

All MR images in this study are owned by University Hospital Frankfurt, Frankfurt, Germany, for research purposes and cannot be made publicly available owing to patient privacy and ethical concerns.

## References

[1] Gandaglia G, Leni R, Bray F, et al. Epidemiology and prevention of prostate cancer. *Eur Urol Oncol*. 2021;4(6):877-892. 10.1016/j.euo.2021.09.006 34716119 10.1016/j.euo.2021.09.006

[2] Nguyen-Nielsen M, Borre M. Diagnostic and therapeutic strategies for prostate cancer. *Semin Nucl Med*. 2016;46(6):484-490. 10.1053/j.semnuclmed.2016.07.002 27825428 10.1053/j.semnuclmed.2016.07.002

[3] Mai J, Abubrig M, Lehmann T, et al. T2 mapping in prostate cancer. *Invest Radiol*. 2019;54(3):146-152. 10.1097/RLI.0000000000000520 30394962 10.1097/RLI.0000000000000520

[4] Grivas N, Lardas M, Espinós EL, et al. Prostate Cancer Detection Percentages of Repeat Biopsy in Patients with Positive Multiparametric Magnetic Resonance Imaging (Prostate Imaging Reporting and Data System/Likert 3-5) and Negative Initial Biopsy. A Mini Systematic Review. *Eur Urol*. 2022;82(5):452-457. 10.1016/j.eururo.2022.07.025 35985901 10.1016/j.eururo.2022.07.025

[5] Turkbey B, Rosenkrantz AB, Haider MA, et al. Prostate imaging reporting and data system version 21: 2019 update of prostate imaging reporting and data system version 2. *Eur Urol*. 2019;76(3):340–351. 10.1016/j.eururo.2019.02.033 30898406 10.1016/j.eururo.2019.02.033

[6] Klingebiel M, Schimmöller L, Weiland E, et al. Value of T2 mapping MRI for prostate cancer detection and classification. *J Magn Reson Imaging*. 2022;56(2):413-422. 10.1002/jmri.28061 35038203 10.1002/jmri.28061

[7] Hepp T, Kalmbach L, Kolb M, et al. T2 mapping for the characterization of prostate lesions. *World J Urol*. 2022;40(6):1455-1461. 10.1007/s00345-022-03991-8 35357510 10.1007/s00345-022-03991-8 PMC9166840

[8] Hoang Dinh A, Souchon R, Melodelima C, et al. Characterization of prostate cancer using T2 mapping at 3T: a multi-scanner study. *Diagn Interv Imaging*. 2015;96(4):365-372. 10.1016/j.diii.2014.11.016 25547670 10.1016/j.diii.2014.11.016

[9] Hilbert T, Sumpf TJ, Weiland E, et al. Accelerated T2 mapping combining parallel MRI and model-based reconstruction: GRAPPATINI. *J Magn Reson Imaging*. 2018;48(2):359-368. 10.1002/jmri.25972 29446508 10.1002/jmri.25972

[10] Griswold MA, Jakob PM, Heidemann RM, et al. Generalized autocalibrating partially parallel acquisitions (GRAPPA). *Magn Reson Med*. 2002;47(6):1202-1210. 10.1002/mrm.10171 12111967 10.1002/mrm.10171

[11] Roux M, Hilbert T, Hussami M, Becce F, Kober T, Omoumi P. MRI T2 Mapping of the Knee Providing Synthetic Morphologic Images: Comparison to Conventional Turbo Spin-Echo MRI. *Radiology*. 2019;293(3):620-630. 10.1148/radiol.2019182843 31573393 10.1148/radiol.2019182843

[12] Gruenebach N, Abello Mercado MA, Grauhan NF, et al. Clinical feasibility and validation of the accelerated T2 mapping sequence GRAPPATINI in brain imaging. *Heliyon*. 2023;9(4):e15064. 10.1016/j.heliyon.2023.e15064 37096006 10.1016/j.heliyon.2023.e15064 PMC10121777

[13] Raudner M, Schreiner MM, Hilbert T, et al. Clinical implementation of accelerated T2 mapping: Quantitative magnetic resonance imaging as a biomarker for annular tear and lumbar disc herniation. *Eur Radiol*. 2021;31(6):3590-3599. 10.1007/s00330-020-07538-6 33274406 10.1007/s00330-020-07538-6 PMC8128819

[14] Vietti Violi N, Hilbert T, Bastiaansen JAM, et al. Patient respiratory-triggered quantitative T2 mapping in the pancreas. *J Magn Reson Imaging*. 2019;50(2):410-416. 10.1002/jmri.26612 30637852 10.1002/jmri.26612 PMC6766866

[15] Li S, Liu J, Zhang F, et al. Novel T2 mapping for evaluating cervical cancer features by providing quantitative T2 maps and synthetic morphologic images: A preliminary study. *J Magn Reson Imaging*. 2020;52(6):1859-1869. 10.1002/jmri.27297 32798294 10.1002/jmri.27297

[16] Draveny R, Ambarki K, Han F, et al. Comparison of T2 Quantification Strategies in the Abdominal-Pelvic Region for Clinical Use. *Invest Radiol*. 2022;57(6):412-421. 10.1097/RLI.0000000000000852 34999669 10.1097/RLI.0000000000000852

[17] Wenzel M, von Hardenberg J, Welte MN, et al. Monoprophylaxis With Cephalosporins for Transrectal Prostate Biopsy After the Fluoroquinolone-Era: A Multi-Institutional Comparison of Severe Infectious Complications. *Front Oncol*. 2021;11:684144. 10.3389/fonc.2021.684144 34178678 10.3389/fonc.2021.684144 PMC8222717

[18] Wenzel M, Theissen L, Preisser F, et al. Complication Rates After TRUS Guided Transrectal Systematic and MRI-Targeted Prostate Biopsies in a High-Risk Region for Antibiotic Resistances. *Front Surg*. 2020;7:7. 10.3389/fsurg.2020.00007 32185180 10.3389/fsurg.2020.00007 PMC7059219

[19] Wenzel M, Welte MN, Theissen LH, et al. Comparison of Complication Rates with Antibiotic Prophylaxis with Cefpodoxime Versus Fluoroquinolones After Transrectal Prostate Biopsy. *Eur Urol Focus*. 2021;7(5):980-986. 10.1016/j.euf.2020.11.006 33358884 10.1016/j.euf.2020.11.006

[20] Wenzel M, Preisser F, Wittler C, et al. Correlation of MRI-Lesion Targeted Biopsy vs. Systematic Biopsy Gleason Score with Final Pathological Gleason Score after Radical Prostatectomy. *Diagnostics (Basel)*. 2021;11(5). 10.3390/diagnostics11050882 10.3390/diagnostics11050882 PMC8155831 34063557

[21] Scherer J, Nolden M, Kleesiek J, et al. Joint imaging platform for federated clinical data analytics. *JCO Clin Cancer Inform*. 2020;4:1027-1038. 10.1200/CCI.20.00045 33166197 10.1200/CCI.20.00045 PMC7713526

[22] Ruprecht O, Weisser P, Bodelle B, Ackermann H, Vogl TJ. MRI of the prostate: interobserver agreement compared with histopathologic outcome after radical prostatectomy. *Eur J Radiol*. 2012;81(3):456-460. 10.1016/j.ejrad.2010.12.076 21354732 10.1016/j.ejrad.2010.12.076

[23] Garcia-Reyes K, Passoni NM, Palmeri ML, et al. Detection of prostate cancer with multiparametric MRI (mpMRI): effect of dedicated reader education on accuracy and confidence of index and anterior cancer diagnosis. *Abdom Imaging*. 2015;40(1):134-142. 10.1007/s00261-014-0197-7 25034558 10.1007/s00261-014-0197-7 PMC4419362

[24] Yamauchi FI, Penzkofer T, Fedorov A, et al. Prostate cancer discrimination in the peripheral zone with a reduced field-of-view T(2)-mapping MRI sequence. *Magn Reson Imaging*. 2015;33(5):525-530. 10.1016/j.mri.2015.02.006 25687187 10.1016/j.mri.2015.02.006 PMC4426240

[25] Liu W, Turkbey B, Sénégas J, et al. Accelerated T2 mapping for characterization of prostate cancer. *Magn Reson Med*. 2011;65(5):1400-1406. 10.1002/mrm.22874 21394778 10.1002/mrm.22874 PMC3079019

[26] Liu L, Yin B, Shek K, et al. Role of quantitative analysis of T2 relaxation time in differentiating benign from malignant breast lesions. *J Int Med Res*. 2018;46(5):1928-1935. 10.1177/0300060517721071 29557239 10.1177/0300060517721071 PMC5991255

[27] Wu L-M, Yao Q-Y, Zhu J, et al. T2* mapping combined with conventional T2-weighted image for prostate cancer detection at 30T MRI: a multi-observer study. *Acta Radiol*. 2017;58(1):114–120. 10.1177/0284185116633916 26917785 10.1177/0284185116633916

[28] Panda A, Obmann VC, Lo W-C, et al. MR fingerprinting and ADC mapping for characterization of lesions in the transition zone of the prostate gland. *Radiology*. 2019;292(3):685-694. 10.1148/radiol.2019181705 31335285 10.1148/radiol.2019181705 PMC6716564

