# Supplementary material for: Value of MRI - T2 Mapping to Differentiate Clinically Significant Prostate Cancer
Source: J Imaging Inform Med. 2024 Jun 26;37(6):3304–15. doi: 10.1007/s10278-024-01150-6 (PMC11612117; doi:10.1007/s10278-024-01150-6)
Supplement: Supplementary file 1 — Supplementary file1 (DOCX 15 KB) [file 10278_2024_1150_MOESM1_ESM.docx]

| Supplementary Information **Table 1 MRI Acquisition Parameters** | | | | | | |
| --- | --- | --- | --- | --- | --- | --- |
|  | T2w tra | T2w sag | T2 cor | T2 Mapping | Diffusion Weighted Imaging | Dynamic Contrast Enhanced Imaging |
|  |  |  |  |  |  |  |
|  |  |  |  |  |  |  |
|  |  |  |  |  |  |  |
| Repetition time (ms) | 7500 | 7500 | 7500 | 5000 | 3700 | 5.08 |
| Echo time (ms) | 101 | 101 | 101 | 10.8-172.8 (10.8 ms intervals) | 58 | 1.77 |
| Field of view (mm) | 200 | 200 | 200 | 220 | 200 | 300 |
| Flip angle (degrees) | 160 | 160 | 160 | 180 | 0 | 13.5 |
| Orientation | axial | sagittal | coronal | axial | axial | axial |
| Scan time (min:sec) | 02:54 | 02:39 | 02:54 | 04:37 | 03:53 | 04:44 |
| b-values (s/mm^2^) | NA | NA | NA | NA | 50, 500, 1000, 2000C | NA |
| Slice thickness (mm) | 3 | 3 | 3 | 3 | 3 | 3.5 |
| Interslice gap | no gap | no gap | no gap | no gap | no gap | 30% |
| In plane resolution x slice thickness | 0.6 x 0.6 | 0.6 x 0.6 | 0.6 x 0.6 | 0.7 x 0.7 | 1.3 x 1.3 | 1.4 x 1.4 |
| (mm x mm x mm) | x 3.0 | x 3.0 | x 3.0 | x 3.0 | x 3.0 | x 3.5 |
| Acceleration factor | 2 | 2 | 2 | 10 | 2 | 2 |
| T2w, T2-weighted; FSE, fast spin echo; NA, not applicable; C, computed | | | | | | |
